# Ascites—III

**Published:** 1909-06-26

**Authors:** 


### Medicine.

### ASCITES—III.

Diagnosis from Other Conditions.
(*Continued.*)

2. *Ovarian Cyst.*—A large unilocular ovarian cyst may give rise to uniform and enormous abdominal distension which may be mistaken for ascites. Sometimes the enlargement of the abdomen is noticed at an early stage to be more on one side than the other, and to have arisen from the pelvis.

The umbilicus may be less deep than normal, and it may even become flush with the surface, but it rarely becomes protruded in the way that is not uncommon in ascites. If the cyst is tense and is lying in contact with the aorta, a well-marked transmitted pulsation may be observed and felt. Similar pulsation does not occur in ascites. A fluid thrill may be obtained only in the anterior part of the abdomen, and not far back in the flanks as in ascites. It may be possible to see the outline of the cyst during respiratory movements, or to feel it.

There is dulness, convex upwards, over the front of the abdomen, and resonance in the flanks; whereas in ascites there is usually dulness in the flanks and resonance in front. Exceptions to this rule have already been discussed. On measuring the abdomen the greatest circumference is generally below the level of the umbilicus, whereas in ascites it is generally at the umbilicus. The latter has often become nearer to the ensiform cartilage than to the pubes, and it is usually nearer to one anterior superior iliac spine than the other; in ascites it is nearer to the pubes than to the ensiform cartilage, and is usually equidistant from right and left anterior superior iliac spines.

A vaginal examination may determine that the uterus is drawn upwards, that its mobility is impaired, that it is of normal size, and that the vagina appears to be lengthened, whereas in ascites the uterus is pushed downwards, is as a rule freely movable, and the vagina appears to be shortened. If paracentesis has been performed the nature of ovarian fluid is often sufficiently characteristic to distinguish it from ascitic fluid. The appearance and characters of ovarian fluid are very variable. In colour it may be clear yellow, greyish, greenish, greenish brown, reddish brown, dark brown, cholocate brown, or even almost black. When dark the colour is usually due to previous hæmorrhage into the cyst as by oozing after a former tapping. The fluid may be clear and watery, but often it is thick, turbid, glairy, mucoid, or slimy. The specific gravity is usually high, from 1020 to 1026, but it may be as low as 1002 or as high as 1050. The reaction is alkaline. There may be a thick deposit on standing, but there is no spontaneous coagulum. Microscopical examination shows red and white blood corpuscles, squamous, columnar, and ciliated epithelial cells, cholesterin crystals, and colloid bodies. A chemical examination discovers serum-albumin, serum-globulin, met-albumin, and salts. The met-albumin is characteristic.

3. *Pregnancy.*—A gravid uterus beyond the fifth month gives rise to general abdominal distension. The umbilicus may be flattened, but it is not as a rule protruded. It is likely to be pigmented, and there will be a dark brown pigmented line running between it and the pubes. The breasts become enlarged, full, and tender; on compressing them fluid may exude from the nipples in some cases as early as the third month. The mammary veins become dilated and prominent, and the areolæ enlarged, pigmented, and moist. There is a violet coloration of the cervix, vagina, and vulva which may start in the cervix in the second or third month. Fœtal movements and uterine contractions may sometimes be seen if the abdomen is carefully observed. On palpating the abdomen the outline of the distended uterus may be felt, and it may present the typical characters of a gestation—namely, a cystic tumour containing both fluid and a solid body, the fœtus. The body, head, and limbs of the fœtus may be distinguished, and fœtal movements and contractions and relaxations of the uterine muscle may be felt. If there is an excess of liquor amnii it may not be possible to make out the above points, and the conditions may readily be mistaken for ovarian cyst or for ascites. A thrill may sometimes be felt; but this is not common unless considerable excess of liquor amnii is present and the maternal parietes are thin.

Important evidence of pregnancy may be obtained by auscultation. Fœtal movements may be heard as thuds, taps, or scratching noises. A uterine souffle may be heard as a soft blowing murmur synchronous with the mother's pulse. It may be just audible at the end of the fourth month, thereafter increasing to term. The souffle is apt to vary from time to time in loudness and in quality; a phenomenon which is probably due in part at least to uterine contractions, and is not usually found in uterine souffle due to other conditions, such as large fibromyomata.

The fœtal heart may be heard. It varies in rate between 120 and 160. It is not heard as a rule before the fifth month. It is the most important of all the signs of pregnancy. It is generally best heard on the left side of the mother's abdomen at a point about half-way between the umbilicus and the centre of Poupart's ligament. Whilst listening for the fœtal heart the mother's radial pulse should be felt at the same time in order to exclude maternal vascular sounds, which would be of the same frequency as the mother's pulse rate, whilst fœtal heart sounds are always faster.

Vaginal examination affords important information in all these cases. In pregnancy, unlike ascites, the cervix uteri is thickened, shortened, and softened. It is frequently patulous, so that the finger tip may be inserted, the membranes being felt, and in some cases even the presentation determined.

Ballottement may be obtained from the fourth to the seventh month. There will also be the usual well-known symptoms of pregnancy, amenorrhœa from the commencement, pain and tenderness of the breasts, nausea and morning sickness, and so on.

